# First Results: Innovative Solar Disinfection Technology for Treated Wastewater that Integrates Materiality, Geometry, and Reflective Panels

**DOI:** 10.3390/ijerph17186523

**Published:** 2020-09-08

**Authors:** Pedro Cisterna-Osorio, Sergio Quijada-Vera, Daniela Ruiz-Duran, Rodrigo Peirano-Cuevas, Pamela Ortiz-Briones

**Affiliations:** Department of Civil and Environmental Engineering, University of Bío Bío, Concepción 378000, Chile; squijada@ubiobio.cl (S.Q.-V.); Dcruizduran@gmail.com (D.R.-D.); r.peirano.cuevas@gmail.com (R.P.-C.); p.ortiz.briones@gmail.com (P.O.-B.)

**Keywords:** solar radiation, disinfection, wastewater

## Abstract

Climate change is having drastic consequences in Chile. The lack of water in various regions is causing environmental impacts on ecosystems, including the decrease in the productive activities of rural economies and the deterioration in the quality of life of the inhabitants that occupy the affected physical spaces. In this paper, we propose a sustainable, low-cost treatment of wastewater and its reuse as an adaptation and mitigation policy, patented in 2019, that consists of a wastewater disinfection system based on solar energy. This system can work in both continuous and discontinuous modes. The water passes through a canal of reflective material in the continuous regime, and in the batch regime, the water remains in the canal. The panels are located parallel to the lateral faces of the canal. These panels concentrate the radiation in the canal through reflection. The trapezoidal geometry of the disinfectant canal deflects the radiation and reflects in the direction of the front walls of the canal, radiating what is returned and vice versa. The fraction of the radiation reflected outside the canal reaches the reflective side panels that return the radiation to the canal. The synergy of these three considerations increases the radiation in the canal area, augmenting the elimination of the bacterial load. In the trapezoidal reflective canal without panels, only 5% of the measured radiation exceeded the atmospheric radiation, eliminating 83% of the coliforms. The incorporation of panels surpassed the atmospheric radiation over 36% of the measured radiations, and the removal of coliforms exceeded 99.7%.

## 1. Introduction

The effects of climate change will affect the dynamics of our society and economy [1]. The magnitude of such impacts will differ depending on the individual capabilities and the collective responses, scientific advances, institutional capacities, and quality of community debate [2,3]. The hydrological cycle generates a series of ecosystem services, including water supply, climate regulation, vegetation, and determining the quality of life for human communities that inhabit the watersheds [4]. The hydrological cycle is especially relevant in zones with a considerable rural population dedicated to agricultural activities [5]. Climate change affects the hydrological cycle causing droughts in Chile and Latin America. Thus, the treatment of wastewater, including disinfection and its subsequent reuse in agriculture, is necessary and contributes to avoiding groundwater contamination and enabling cost-savings for nutrient removal [6].

The disinfection treatments are classified as conventional, advanced, and natural processes. The natural processes include wastewater system stabilization ponds and constructed wetlands [7].

Chlorination is the most conventional disinfection treatment. However, the toxicity of the by-products generated by chlorination in wastewater should be considered. The regulatory agencies insist on the use of ecologically sustainable alternatives [8]. The availability and costs constitute a problem. Thus, a cleaner and cheaper technology for the disinfection of water is necessary in developing countries [9].

The disadvantages of disinfection by chlorination promoted and potentiated disinfection by ultra violet (UV) light. Currently, UV disinfection is a competitive alternative to chlorination [10].

For the emerging UV disinfection solution of solar radiation, the albedo (a) is a relevant factor. The albedo (a) is defined as the ratio of reflected (upwelling) irradiance (Iu) with respect to the incident (downwelling) irradiance (Id), and is generally applied only for horizontal surfaces [11]. The reflectivity may change depending on the incident angle of the irradiance on a non-horizontal surface [12].

a = Iu/Id.(1)

Materials with high albedo and emittance attain lower temperatures when they are exposed to solar radiation, reducing the transmission of heat to the environment [13]. Research showed that irradiance measured on a horizontal surface is not proportional to irradiance on a vertical surface. The relation between the two depends on the orientation of the vertical surface, zenith angle, and wavelength. At short ultra violet B (UVB) wavelengths, the surfaces directed toward the solar azimuth received their maximum irradiances much closer to solar noon than the maxima for longer wavelengths [14]. Bacterial inactivation in UV techniques, such as solar disinfection (SODIS), happens because photons may increase the amount of internal reactive oxygen species (ROS), such as hydroxyl radicals (OH) and superoxide radicals (O_2_−). The attacks of ROS on different components of the cells are a primary source of oxidative damage to cells. Photons may damage the two essential enzymes of the defense system against intracellular oxidative processes, catalase and superoxide dismutase [15]. 

A survey of pilot-scale studies investigating UV light as a combined sewer overflow (CSO) disinfectant, suggests that the correct application of UV light irradiation is an effective alternative to chlorination for CSO. The process of disinfecting with UV light appears to be strongly dependent on water quality. Thus, pretreatment of CSO before disinfection is a prerequisite to ensure UV light effectiveness [16]. As an alternative technology, UV disinfection is gaining increasing popularity as it has been shown to effectively inactivate a wide range of pathogens, including the most problematic waterborne parasites, such as *Cryptosporidium* and *Giardia* [17].

Approximately 5% of solar radiation is ultraviolet, with the maximum reported value around 5 mW/cm^2^. The range of UV radiation lies between 100 and 400 nm and is divided into three categories: ultra violet A (UVA) (315–400 nm); ultra violet B (UVB) (280–315 nm); ultra violet C (UVC) (200–280 nm). UVC radiation is absorbed by the ozone layer as well as a proportion of the UV-B radiation; therefore, UVA and a portion of UVB radiation reach the earth’s surface [18]. Although ultraviolet light is not yet widely used as a chemical disinfectant, like chlorine and ozone, there is considerable evidence that the UV method may become a reliable alternative to disinfecting wastewater [19].

Human beings are pathogen incubators. Thus, if their metabolic wastes are treated in a chlorine wastewater treatment plant, which is not effective against *Cryptosporidium* spp., or it is used at doses that have a minimal impact on *Giardia *spp., the wastewater treatment plants can become major sources chlorine-resistant pathogens. These protozoan pathogens are extremely sensitive to UV, therefore, the advantages of UV in protecting water sources are evident [20].

The innovative system developed in this paper is a type of natural disinfection technology. Constructed wetlands reduce the bacteriological load. The mechanisms to reduce bacterial populations include physical and chemical processes, such as the release of oxygen from plants and biological mechanisms, such as natural death, predation, and the action of antibiotic substances released by macrophytes [21]. Tannic acid and gallic acid are secreted from the roots of many aquatic plants and disinfect their surroundings. In addition, the development of populations of bacteria in the rhizosphere with antibiotic properties (e.g., *Pseudomonas*) may also contribute to the removal of *Escherichia coli* [22].

Maturation ponds are generally oxygenated through photo-synthesis due to the relatively high optical clarity of effluents received from the optional pond, wetlands, or other wastewater treatment. It is assumed that an increase in dissolved oxygen would result in an increase in ROS formation and a corresponding increase in photo-oxidation [23].

The maturation ponds possess a depth range of 0.6 m to 0.8 m. This condition is required because levels nearer to the surface of these ponds can receive enough solar radiation for bacterial inactivation. Light intensity decays as depth increases [24]. The presence of algae in natural wastewater treatment systems can contribute appreciably to disinfection [25]. 

A technology based on solar energy is SODIS, which requires a threshold of 500 W/m^2^. The real efficacy of SODIS was tested by inactivating the total coliforms (TC) of *Escherichia coli* (*E. coli*). The exposure period was critical; in 2 days, complete microbial inactivation was achieved [26]. The efficiency of solar disinfection (SODIS) for the inactivation of the total coliforms and *Escherichia coli* in drinking water was tested in rural communities of the Guachochi Municipality, State of Chihuahua, Mexico. The test resulted in complete disinfection when placing transparent water bottles in the sunlight for the whole day, and the water temperature reached 50 °C [27].

Based on preliminary work, a solar disinfection unit was designed and built. The unit was evaluated by the “*Centro Panamericano de Ingenieria Sanitaria y Ciencias del Ambiente (CEPIS)*” in Lima, Perú. In 30 min in midday and light sunlight, the unit eliminated more than 99.99% of bacteria contained in highly contaminated water samples from two wastewater treatment plants [28]. 

An innovative solar water pasteurizer was developed to directly heat the water by solar radiation using a “Parabolic Trough Concentrator” [29]. The enhanced reactor reduced the exposure time required to achieve the lethal UVA dose, as compared to a compound parabolic concentrator (CPC) system with a concentration factor of 1.0. Doubling the lethal UVA dose prevented the need for a period of post-exposure dark inactivation and reduced the overall treatment time. By using this SODIS reactor, the disinfection can be carried out with a reduced exposure time. [30].

For a solar disinfection (SODIS) reactor of methacrylate, a tube is placed along the linear focus of the CPC and is mounted at a 37° inclination to treat 25 L of water. For strong sunlight, complete bacterial inactivation occurred in under 6 h, with water temperatures less than 40 °C. Under cloudy conditions and low solar radiation, the water was disinfected in 7 h with water temperatures greater than 50 °C. Regrowth of bacteria occurred after 24 h following solar disinfection [31].

A presence/absence detection method was developed to analyze *lettuce* leaves sampled 24 h after watering for the detection of *E. coli.* The absence of E. coli on most lettuce samples after irrigation with solar radiation-disinfected effluents (26 negative samples/28 samples) confirmed an improved safety of irrigation practices due to solar treatment [32]. The photo-reactivation processes were compensated entirely by solar disinfection within a 120 min exposure time [33].

Several technological attempts at disinfection based on solar radiation have been proposed. However, these treatments are not sufficiently functional. This manuscript presents the initial development of a new technology based on the use of solar radiation to disinfect treated wastewater, with a low cost of investment and operation. This technology will allow the reuse of wastewater and was patented in 2019 [34]. 

The purpose of this manuscript is to expose the early results of this new technological development, which is cheap, easy to build, operate, and maintain, in the disinfection of treated wastewater. Preliminary results are shown to support it. 

A comparative analysis of scenarios that progressively integrate the basal components of this technology, as well as the materiality, geometry, and reflector panels, is shown, using the radiation rate obtained measuring the solar radiation in the disinfection canal, the atmospheric solar radiation, and the elimination of total coliforms as the principal quantitative indicators.

## 2. Materials and Methods 

Experimental work was conducted to obtain information regarding the variables and relations informing the dynamics of the solar disinfection system. The process depended on the UV radiation, structure, configuration, and total coliform contents present in the incoming and exiting wastewater.

Two different disinfection system were analyzed and compared (with and without panels), and the applied methodology was equal for each experimental case. In both cases, the total coliform (TC), was measured by means of the most probable number (MPN) for different hydraulic residence times. In the case of radiation, a spatial analysis was conducted, measuring this variable in different points of the canal according to the monitoring template.

### 2.1. Disinfection System 

**Figure 1:**
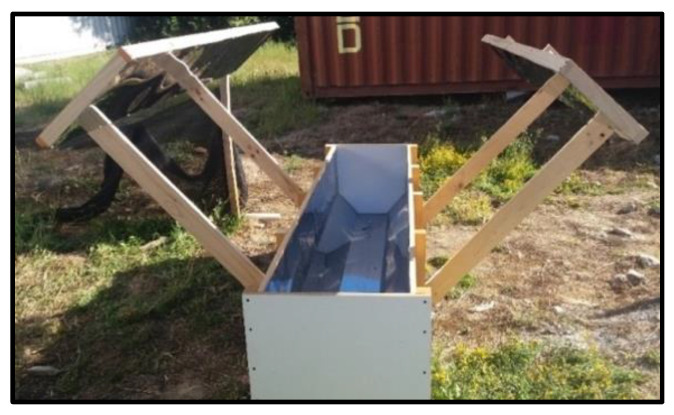
Canal and panels.

The equipment used is described as follows (Figure 1).

The proposed design is based on a simple structure, with geometry and materiality that allows for an increase in the radiation that acts on the water. This result was achieved by means of the advantages of materiality, geometry, and complementary elements such as panels. The canals were designed using a trapezoid cross-section, causing a redirection in a fraction of the sun’s rays that remained intact and reached the base of the canal, and thus a fraction crossed the volume of water again.

**Figure 2:**
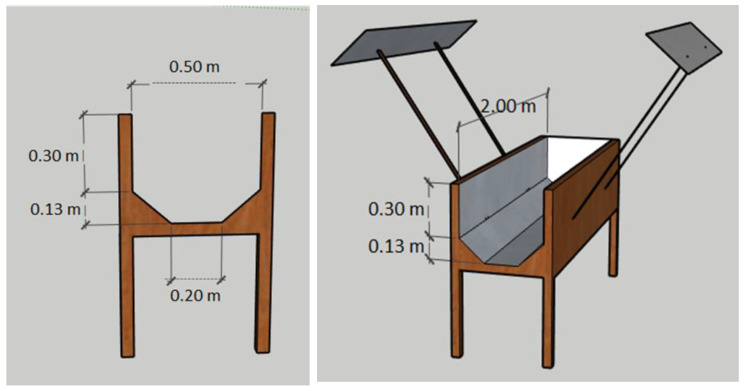
The disinfection equipment dimensions.

Wood structures supported the canals, using pine tree boards of 50 × 75 mm and fastened plates of oriented strand board (OSB). The width of the canal was 500 mm, and the total length was 2000 mm. These structures supported the weight of the canal water. See Figure 2.

Stainless steel was selected as a suitable alternative material with reflection qualities and easy acquisition. Stainless steel shows a low spectral reflectance of around 50–60% in the visible wavelength range, less than other metals, such as aluminum and copper. Therefore, the results can reasonably be expected to improve. Additionally, it is feasible to improve the reflectivity of the stainless-steel surface efficiently by adding a high reflection coating [35].

The installation of the panels had an inclination of 41° in relation to the horizontal. Thus, the panels parallel to the canal had an inclined section of trapezoidal geometry. The installation of the panels is illustrated in Figure 2. The panels were built with pine boards of 50 × 25 mm and were supported by pine sections of 100 × 25 mm. The dimensions of the panels were 2000 × 50 × 300 mm (Figure 2).

### 2.2. Analytical Methods and Monitoring Parameters

The analytical methods and monitoring parameters are described below.

#### Description of Equipment and Ultraviolet Radiation Measurement

The ultraviolet radiation (UVA and UVB) was measured during the experiments. To measure the UVA and UVB, the PUV-360 model was used (see Table 1, Technical Specifications). 

The radiation meter chosen to monitor the ultraviolet radiation (UVA and UVB) during the experiments was acquired from the industrial/agricultural instrumentation company, INSUMAC. The PUV-360 model has a microprocessor circuit that provides high reliability and durability.

### 2.3. Experimental Methodology

The activities included in the experimental methodology are described below.

#### 2.3.1. UV Radiation Determination in Disinfection Canal

**Figure 3:**
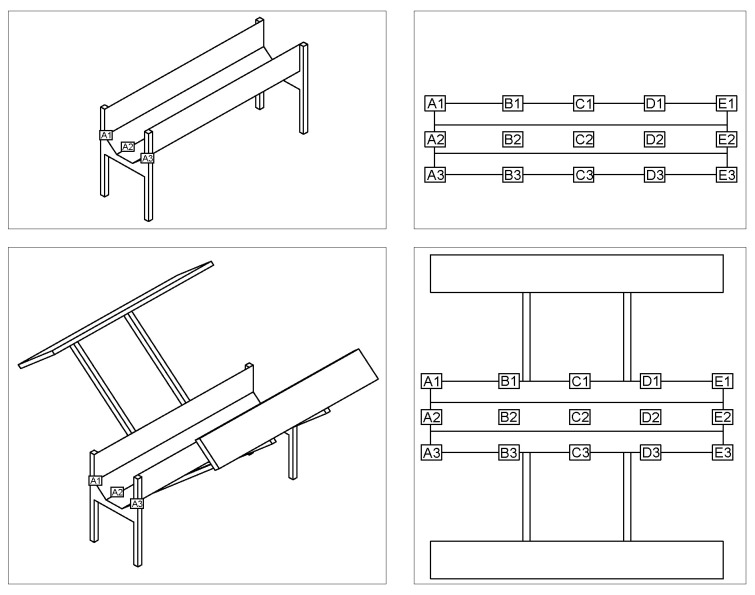
The measuring point template.

The radiation was measured in the solar disinfection system. According to Figure 3, this was established by a template of points located in the contour zones and interiors. The innovative disinfection system was installed in dependencies of the University of Bío Bío, Concepción, Chile.

Four radiation measurements were made in the disinfection canal according to what is indicated in Figure 3, quantity and similar measurements were made for the disinfection canal with side panels, allowing the comparative evaluation to be conducted. These measurements were made with water. 

#### 2.3.2. Normalisation of the UV Radiation, Radiation Rate (RR)

The measurements made in this work were under real conditions, which corresponded to the weather conditions of the day; the variable that was monitored and measured was the UV radiation. Therefore, this was not reproducible, nor was it manageable. For each day of measurement, and throughout the days, the atmospheric radiation had different values. Therefore, we cannot compare the measurements between them as the environmental conditions determined the radiation values obtained. An indicator was created to allow the comparison of measurements from different days in a consistent manner.

As the solar radiation was different every day, and this was random and could not be controlled or reproduced because it was a natural variable, a parameter radiation rate was defined. This parameter allowed us to compare the radiation measurements (Rij) corresponding to each point of the template of monitoring and links the radiation measured in the template at a position (Xij), concerning the atmospheric radiation (Ra).

Rij = Ra, Rij/Ra, RR = 1, for Rij > Ra, RR > 1 and for Rij < Ra, RR will be less than 1.
Therefore: RR = Rij/Ra, i = A, B, C, D, E; j = 1,2,3.

#### 2.3.3. Determination of the Total Coliform (TC)

The solar disinfection system was fed with wastewater treated by activated sludges from the plant of Hualqui, province of Concepción, whose coordinates are U.T.M. 686,393.79 m E; 5,905,081.35 m S Chile.

The treated sewage was deposited on the canal and subjected to disinfection using this technology based on exposure to solar radiation. A sample was collected to measure the total coliforms. This activity was performed in a time range of 225 min, samples were taken each 45 min. The wastewater treated without disinfection corresponded to time 0. After 225 min of exposure to UV radiation, the canal was emptied and washed.

A sample of the residual treated water was taken at the beginning of the experiment using a 250 mL closed glass with a top. This closed glass was submerged between 5 and 10 cm. Once submerged, the glass was opened, allowing the entrance of water. Then, the glass was pulled out of the water and introduced to a cooler. This process was repeated for the following samples and according to the predefined residence times. Finally, the samples were sent to the laboratory for analysis. 

The samples collected for microbiological analysis were kept at a low temperature in a cooler and analyzed on the same day they were collected. The multiple tube technique was used for the total coliform counts and most probable number (MPN) determination [36]. 

The treated wastewater was disinfected under two situations. (A) In the disinfection canal and (B) in the disinfection canal with reflective side panels. Two experiments of coliform abatement were conducted considering a residence time range from 0 to 225 min. In both cases, two measurements were made.

The initial coliform condition, TCo, in the wastewater that was subjected to disinfection, was measured at different hydraulic residence times, and, with this data, we estimated the efficiency of the coliform removal.

Elimination = (TCt − TCo)/TCo

Such that,

TCt = MPN to time t
TCo = MPN to time 0.

## 3. Discussion and Results

### 3.1. Canal Radiation

The radiation was measured at different points of the disinfection canal (shown in Figure 3). Here, the radiation was determined by the actual atmospheric conditions. The reflection capacity of the canal is based on the materiality and trapezoidal geometry. Both conditions generate internal reflection, and, according to previous research, processes such as reflection at surfaces within the canal affect the global radiation, impacting the performance of the UV canal [37]. 

Table 2 shows the results of the radiation obtained according to the templates in Figure 3.

In the case of the canal without panels, we observed that the radiation measured at 57 of the 60 measurements points, corresponding to the four experiments, was less than or equal to the atmospheric radiation. The previous figure indicates that the effects of materiality and geometry are not enough to reach the value of atmospheric radiation.

**Figure 4:**
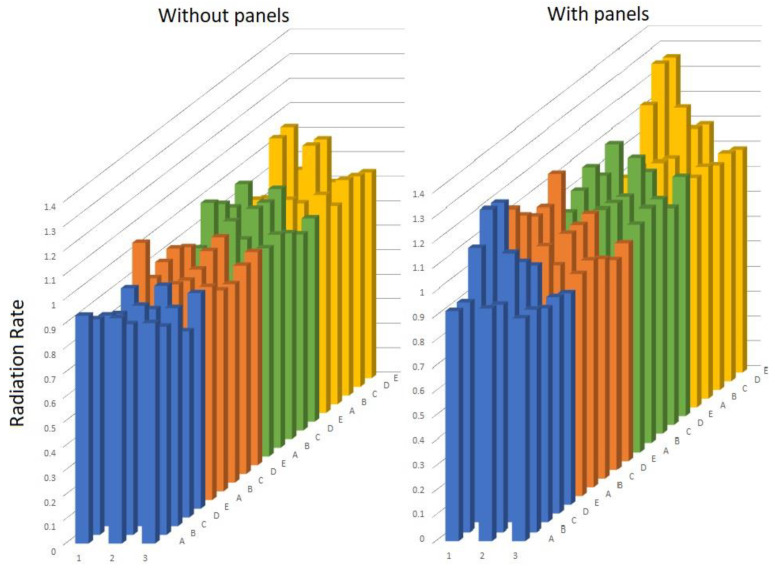
Radiation rate in canals with and without panels.

In the situation with panels, the differential impact of the panels on the resulting radiation at the different monitoring points of the canal is evident. We verified that the radiation measured at 22 of the 60 monitoring points (from Figure 4) exceeded the impact of the side panels on the radiation, and more of the 37% of the measurement points exceeded the value of the atmospheric radiation, unlike the canal without panels. The radiation ratio values of the canal with panels were higher than the canal values without panels. 

### 3.2. Average Values and Comparison of the Radiation Rate with and without Panels

Below are the average radiation rates with and without panels. A comparative analysis of the radiation behavior for the two scenarios is shown. The difference between the average radiation rate with and without panels, for each measured point of the template atmospheric radiation, demonstrated that the effect of the panels increased the number of displacements of the radiation between the respective wall of the canal and the front panel, and approached and exceeded the atmospheric radiation.

Table 3 shows the results obtained for the radiation rate for the canal without panels and with side panels. Table 3 provides the information for graphs 4 and 5.

Figure 4 depicts a graphic comparison of the monitored radiations for both the radiation canal with and without side panels, in order to assess the impact caused by them.

When analyzing Table 3, for the disinfection canal without panels, most of the RR values are less than 1 and vary between 0.8 and 0.9, which implies that the radiations measured in the zone of the disinfection canal were less than the atmospheric radiation value, reaching 80% to 90% of the value.

The difference in the disinfection with panels was that in the four measurements, the RR exceeded the value of 1 in a considerable percentage, which implies that the radiation reached in the measurement template was very near or above the value of the atmospheric radiation. This effect was due to the panels.

From Figure 4, the impact of the side panels caused an increase in the radiation rate, and more than 37% of the measurement points exceeded the value of 1 for the radiation rate. The values of the radiation rate of the canal with panels are higher the canal values without panels. 

### 3.3. Average Values and Comparison of the Radiation Rate with and without Panels

**Figure 5:**
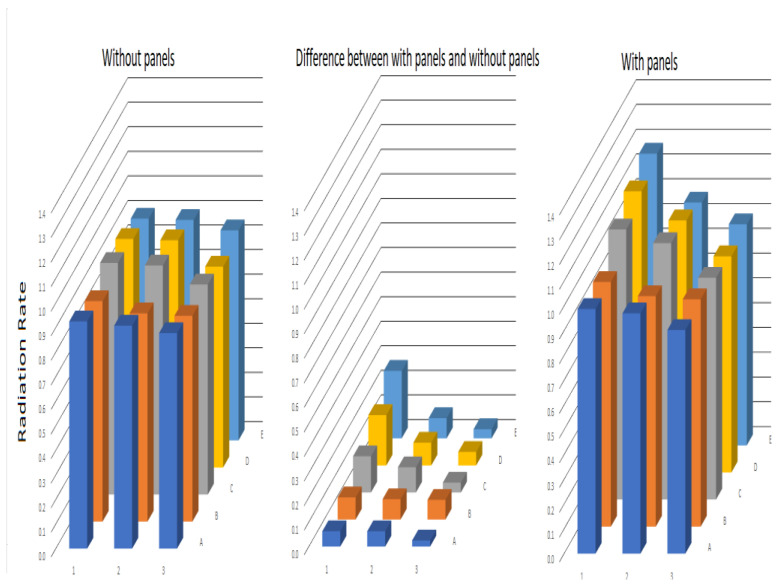
Statistical representation of the behavior of the radiation rate.

Below are the average radiation rates with and without panels. A comparative analysis of the radiation behavior for the two scenarios is shown. The differences between the average radiation rate with and without panels, for each measured point of the template are shown in Figure 5.

We observed that average values for all radiation rate measurements in the canal with side panels were higher than in the canal without panels, showing that the panels reflected and returned the energy coming from the sidewalls of the canal, increasing and concentrating the radiation in the disinfection zone.

This raises the search for an adequate material for the panels and the canals. The thin-film optics, which are based on the phenomenon of light interference [38], are widely used in high reflection applications [39] and bandpass filters [40] in the optical, electronic, and solar industries. 

The high reflection coatings significantly improve the surface reflectivity in a specific wavelength range. A coating can be designed as a long or short pass filter or a mirror with a specific reflectivity [41]. These favorable results on the increase in radiation in the canal with panels are a low-cost solution, adequate for rural communities with low populations. If we incorporate the related technological developments, the field of application of this technology can be extended to industry and large populations.

### 3.4. Incidence of Panels on the Total Coliforms Elimination in the Disinfection Canal

Below in Table 4 is shown the elimination of the total coliforms in the disinfection canal without side panels, and the total coliform elimination achieved in the case with reflective material and trapezoidal geometry. The material reflected the solar radiation, and the geometry caused a redirection of the solar radiation, increasing the number of displacements of the radiation between the respective walls of the canal.

### 3.5. Elimination of Coliforms in Disinfection Canal with Reflective Side Panels

The high rate of elimination of coliforms achieved is explained by the use of a reflective material, a trapezoidal geometry, plus the reflective side panels, which managed to improve the efficiency of the previous configuration considerably. Experiment 1 worked with a height of 11 cm of the water column and Experiment 2 with 22 cm, however, in both cases, the elimination of coliforms exceeded 99%.

Table 5 shows a significant elimination of the total coliforms in cases where the disinfection canal was assisted with side panels. In both situations, the wastewater had a total coliform concentration of 1,600,000 NMP/100 mL, and after 225 min, they reached a concentration of 9400 and 4300 NMP/100 mL for water column heights of 11 and 22 cm, respectively.

**Figure 6:**
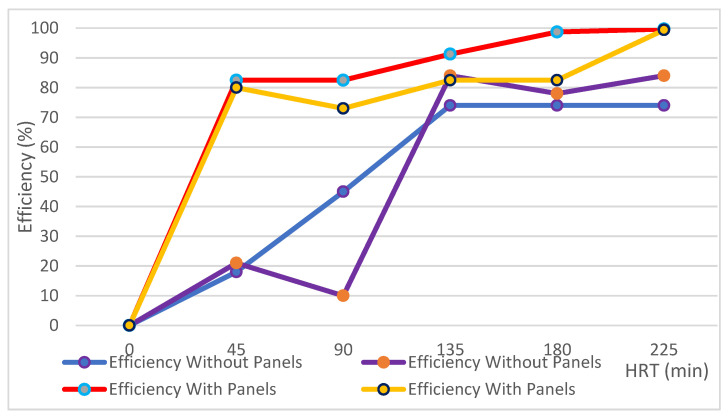
The compared elimination efficiencies of the disinfection canals.

With the results obtained, a comparative analysis of the elimination of total coliforms for the five hydraulic residence times (HRT) in the disinfection canal was performed. We observed that without panels, 80% total coliform elimination was achieved. Then, for the same HRTs of the panel disinfection canal, total coliform elimination was achieved above 99% at 225 min, surpassing the configuration that did not include the assistance of side panels (Figure 6).

In previous research, the impacts of parameters under direct exposure to the sun were studied: depths of 10, 20, and 30 cm and container colors of white and black. The maximum removal of total coliforms was found to be 92.95% at 10 cm depth for the white container [42], and a similar result with the reflective canal worked for the same depth range and grey color.

These findings of photo-biological inactivation of coliforms could be applied in reducing the hazards of waterborne pathogens in an environmentally sound and low-cost system, being an alternative to the glass tube reactor that includes compound parabolic collector (CPC) technology [30]. The reflective canal with panels can also work in a continuous regime.

Other researchers studied the effect of dark treatment on the photoreactivation of *Escherichia coli* in a tertiary effluent disinfected by UV, maintained in darkness, and then put under photoreactivating light. The experimental results for water samples kept in darkness are shown below, Table 6. 

This work suggested that photoreactivation can only be weakened but cannot be controlled entirely even after being kept in darkness for 24 h [43]. In most cases, these treated waters will be used in irrigation, so photoreactivation is an aspect to consider. However, this technology, as well as other UV-based options, has validity and competitiveness.

Finally, from Figure 6, the panels significantly increased the efficiency of total coliform removal. The implementation of panels acted efficiently in the reflection of sunlight, and caused a positive impact on the removal of total coliforms at a lower cost. 

### 3.6. Elimination of Coliforms in Disinfection Canal for Effluent from Artificial Wetland

In Table 7, we show the results previously obtained for the disinfection of treated wastewater in an artificial wetland high performance [44] with a horizontal subsurface flow. This treatment sequence is the most indicated for small communities, due to its operational simplicity, low costs, and its environmental sustainability.

**Figure 7:**
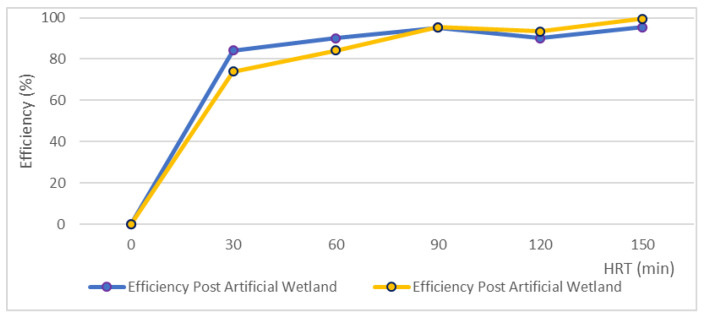
Compared elimination efficiencies of the disinfection canal.

Figure 7 shows a significant elimination of the total coliforms for the disinfection canal without side panels; the total coliform concentration of 5000 NMP/100 mL was within the range of an effluent from an artificial wetland.

In summary, the proposed disinfection system is viable in discontinuous flows as well as continuous flows, which constitutes a significant advantage due to its functionality and adaptability to different scenarios, and elasticity as it serves a wide range of flows.

## 4. Conclusions

The appropriate combination of materiality and geometry in the wastewater disinfection canal achieved an average radiation equivalent to 87% of the atmospheric radiation and a total coliform elimination above 75% for a time of 225 min. The incorporation of lateral reflective panels to the metallic reflector and trapezoidal canal achieved average radiation equivalent to 97% of the atmospheric radiation and increased the elimination of total coliforms to 99%.

The incorporation of side reflective panels substantially improved the results of the disinfectant canal. From the results obtained empirically and the literature review, we concluded that this technology has enormous potential to incorporate improvements based on the knowledge and developments, maintaining its structure as a reflective canal with specific geometry and reflective panels.

We concluded that the combined use of alternative technologies in rural populations or low population communities solves the issue of wastewater treatment and reuse in a sustainable way and at a low cost.

## Figures and Tables

**Table 1:** The PUV-360 reflectometer technical specifications.

Technical Specifications	
Monitor/UV sensor spectrum	LCD size: 28 × 19 mm/Bandpass from 290 nm to 390 nm
Measuring/Resolution Ranges	range 1:2000 uW/cm^2^: 1999 uW/cm^2^ × 1 uW/cm^2^ range 2:20 mW/cm^2^: 19.99 mW/cm^2^ × 1 mW/cm^2^
Precision	(4% of the read + 2 dig.). Calibration was performed under UVA light and compared with a standard reference field light meter less than 3 V/M and frequency less than 30 MHz
Sensor structure/Sample time	Photo UV sensor with cosine correction filter/1 s approx.
Off/Weight	Auto power off saves battery life/190 g/0.2 LB
Humidity (HR)/Temperature Operation	Less than 85% HR/0 °C to 50 °C
Dimensions/Power Supply	210 × 49 × 40 mm/4 AAA batteries.

**Table 2:** Radiation in the canal without and with panels.

	UV Radiation in the Disinfection Canal (mW/cm^2^)											
	Without Panels						With Panels					
	Data	A	B	C	D	E	Data	A	B	C	D	E
Measure 1	1	4.4	5.26	5.18	4.02	4.18	1	4.51	4.58	5.6	6.26	6.2
	2	4.29	4.98	5.05	4.05	3.96	2	4.79	4.7	5.55	4.98	4.86
	3	4.31	4.28	4.34	4.18	4.22	3	4.5	4.55	4.43	4.46	4.35
		Ra = 4.95						Ra = 4.91				
Measure 2	1	3.91	4.6	4.42	4.2	4.45	1	4.4	4.61	4.9	4.6	5
	2	4.42	3.9	4.33	4.3	4.37	2	4.42	4.4	4.33	4.9	4.47
	3	3.93	4.01	3.88	3.7	3.81	3	4.18	4.3	4.3	3.98	4.4
		Ra = 4.61						Ra = 4.58				
Measure 3	1	5.69	4.69	4.9	4.98	4.82	1	5.05	4.81	4.63	4.62	5.07
	2	4.77	4.65	4.72	4.92	5.02	2	4.42	3.9	4.33	4.3	4.37
	3	4.7	4.47	4.38	4.63	4.72	3	3.93	4.01	3.88	3.7	3.81
		Ra = 5.42						Ra = 4.4				
Measure 4	1	5.04	4.78	4.66	4.52	4.87	1	4.72	4.75	5.64	6.29	6.2
	2	5.03	4.7	4.87	4.64	4.96	2	4.79	4.7	5.55	5.18	4.96
	3	4.88	4.6	4.85	4.16	4.77	3	4.55	4.59	4.43	4.46	4.35
		Ra = 5.44						Ra = 5.14				

**Table 3:** Radiation rates in canal radiation without/with panels.

	Radiation Rate											
	Without Panels						With Panels					
	Data	A	B	C	D	E	Data	A	B	C	D	E
Measure 1	**1**	0.87	0.84	1.05	1.06	0.85	**1**	0.92	0.93	1.14	1.27	1.26
	**2**	0.87	0.82	1.02	1.01	0.8	**2**	0.98	0.96	1.13	1.01	0.99
	**3**	0.89	0.81	0.88	0.86	0.84	**3**	0.92	0.93	0.9	0.91	0.89
Measure 2	**1**	0.85	1	0.96	0.91	0.97	**1**	0.96	1.01	1.07	1	1.09
	**2**	0.96	0.85	0.94	0.93	0.95	**2**	0.97	0.96	0.95	1.07	0.98
	**3**	0.85	0.87	0.84	0.8	0.83	**3**	0.91	0.94	0.94	0.87	0.96
Measure 3	**1**	1.05	0.87	0.9	0.92	0.89	**1**	1.15	1.09	1.05	1.05	1.15
	**2**	0.88	0.86	0.87	0.91	0.93	**2**	1	0.89	0.98	0.98	0.99
	**3**	0.87	0.82	0.81	0.85	0.87	**3**	0.89	0.91	0.88	0.84	0.87
Measure 4	**1**	0.93	0.88	0.86	0.83	0.9	**1**	0.92	0.92	1.1	1.22	1.21
	**2**	0.92	0.86	0.9	0.85	0.91	**2**	0.93	0.91	1.08	1.01	0.96
	**3**	0.9	0.85	0.89	0.76	0.88	**3**	0.89	0.89	0.86	0.87	0.85

**Table 4:** Elimination of coliforms in the disinfection canal without panels.

	Total Coliforms (TC) Elimination without Panels			
	Experiment 1		Experiment 2	
Time (Min)	TC (MPN/100 mL)	TC Elimination (%)	TC (MPN/100 mL)	TC Elimination (%)
0	170,000		280,000	
45	140,000	17.6	220,000	21.4
90	94,000	44.7	260,000	7.1
135	43,000	74.7	43,000	84.6
180	43,000	74.7	61,000	78.2
225	43,000	74.7	43,000	84.6

**Table 5:** Elimination of coliforms in the disinfection canal with panels.

TC Elimination with Panels				
	Experiment 1		Experiment 2	
Time (Min)	TC (MPN/100 mL)	TC Elimination (%)	TC (MPN/100 mL)	TC Elimination (%)
0	1,600,000		1,600,000	
45	280,000	82.5	280,000	82.5
90	280,000	82.5	480,000	70.0
135	140,000	91.3	280,000	82.5
180	21,000	98.7	220,000	86.3
225	4300	99.7	9400	99.4

**Table 6:** Darkness and photo reactivation.

E (mJ/cm^2^)	Time (h)	Percentage (%)
5	6	31.1
5	24	0.4
20	6	10
20	24	0.3

**Table 7:** Elimination of coliforms in the disinfection canal with panels.

TC Elimination Post Artificial Wetland				
	Experiment 1		Experiment 2	
HRT (Min)	TC (MPN/100 mL)	TC Elimination (%)	TC (MPN/100 mL)	TC Elimination (%)
0	5000		5000	
30	790	84.2	1300	82.5
60	490	90.2	790	84.2
90	240	95.2	230	95.4
120	490	90.2	330	93.4
150	230	95.4	23	99.5
